# Supplementary material for: Are carbon emissions trading and green financial instruments synergistic? -Comprehensive quantitative research based on content analysis
Source: PLoS One. 2024 Mar 7;19(3):e0298601. doi: 10.1371/journal.pone.0298601 (PMC10919601; doi:10.1371/journal.pone.0298601)
Supplement: S1 Data — (ZIP) [file pone.0298601.s001.zip › Data/地级市Z_F工作报告.zip!/║╙─╧/░▓╤⌠╩╨/└┤╫╘╣1⁄2╓┌║┼í╛┬φ┐╦╩2╛▌═°í┐_2016.docx]

各位代表：

    现在，我代表市人民政府向大会报告工作，请予审议，并请各位政协委员和其他列席人士提出意见。

    一、2015年及“十二五”工作回顾

    2015年，面对复杂严峻的经济形势和近年少有的困难局面，在省委、省政府和市委的正确领导下，我们坚持转中求进、改革创新，多策并举、精准发力，经济社会平稳健康发展，较好完成了市十三届人大二次会议确定的各项目标任务。初步统计，全市生产总值1672亿元，增长7.2%；规模以上工业增加值727.8亿元，增长5.6%；一般公共预算收入100.3亿元，增长5.3%；固定资产投资1682亿元，增长16.3%；社会消费品零售总额592.4亿元，增长12.1%；城乡居民收入分别达到28128元和12382元，均增长8%。我们重点抓了以下工作：

    （一）稳增长保态势，努力保持经济平稳健康发展。把稳增长保态势作为全局工作的突出任务，出台实施促进经济平稳健康发展30条、财政支持稳增长28条，制定关于大众创业万众创新、融入“一带一路”等8个专项方案，经济运行稳中趋好。

    着力扩大投资。实施“661工程”“十大建设工程”，成立29个重大项目领导小组和5个督导服务组，集中精力抓项目、扩投资、稳增长。安彩高科LNG液化工厂、振晨重工千米钻机、联达数控精密锻造等148个项目开工建设或投产。争取中央和省政策性资金项目451个、资金26.9亿元。183个省、市重点项目完成投资648亿元，超计划进度57个百分点。

    加强企业帮扶。制定行业企业帮扶解困专项工作方案，成立5个帮扶重点行业和10个帮扶重点企业专题工作组。清理规范涉企收费，落实中小微企业政策，着力帮助企业降负担、增活力、拓市场。规模以上工业企业主营业务收入3320.5亿元，利润总额151.9亿元。

    强化要素保障。分别设立2.5亿元的私募股权投资基金、产业发展基金、创业投资基金、政府和社会资本合作专项开发性基金。深入开展“扩融资保资金行动计划”“融资服务企业百家行”活动。51家企业在新三板等各类股权交易市场挂牌。金融机构各项贷款余额1009.6亿元，新增130.8亿元，贷款增量创历史新高。

    促进房地产发展。出台《关于促进房地产市场持续健康发展的若干意见》，实施购房补贴政策，鼓励住房消费，有效减少了库存、拉动了消费。全年商品房成交31199套，增长69%；房地产开发投资增长29.7%，居全省第一位。

    （二）调结构促转型，不断加快发展方式转变。把调结构转方式作为主攻方向，努力在优化结构中稳增长，在创新驱动中促转型。

    推动产业结构调整。实施“工业转型升级400”，努力优存量、扩增量，产业结构持续优化。全市高成长性制造业完成投资增长23.1%，占工业投资的50%，提高4个百分点。大力发展商贸物流，加快培育新业态新模式。万达广场开业。万庄物流园、内黄果蔬城等有序推进。电商大厦建成投用。大数据云计算中心正式开通。阿里巴巴安阳产业带上线运行。全国工业电子商务区域试点挂牌。全年接待国内外游客3431万人次，增长15.6%；旅游总收入253.4亿元，增长28.3%。

    促进农业稳产增效。实施现代农业五大工程。新建高标准粮田31万亩，粮食总产227.4万吨，实现“十三连增”。农业产业集群总收入252亿元，10亿元集群达到15个。累计建成农业园区146个、80.3万亩。新增农业合作社106家、家庭农场120家。农产品质量安全监管体系不断加强。

    加强科技创新。制定出台《关于强力推进科技创新引领产业转型升级的若干政策》《发展众创空间推进大众创新创业的实施意见》，与清华大学签订全面合作协议，同西安交大、合肥工大等高校合作更加紧密。实施对外科技合作项目48个。新增院士工作站3个、市级研发中心18家。新培育众创空间6家。

    推进绿色发展。落实生态市建设规划，营造林19万亩，新增城市绿地71.4万平方米。通过国家园林城市届满复审。深入推进大气污染长效治理，大力实施蓝天工程，累计投入13.5亿元，顺利解除环保挂牌督办和区域限批，市区空气质量优良天数达标率明显提升。

    （三）强基础抓统筹，持续增强综合支撑能力。坚持长短结合，加强发展平台和基础设施建设，支撑能力持续增强。

    完善科学发展载体。推动产业集聚区和服务业“两区”提质转型创新发展。精品钢、装备制造2个千亿元主导产业集群和煤化工、陶瓷、食品医药、纺织服装、电子信息、有色金属等6个百亿元特色产业集群加快推进。全市产业集聚区完成投资679.6亿元，增长23%；实现工业总产值1629亿元，增长15.8%。

    强化基础设施支撑。西北绕城高速公路开工建设。国道341加快推进。15条新建、改建城市道路基本竣工。新增100辆纯电动公交车。城市公共自行车服务系统建成投用。南水北调配套工程基本完工。引彰入羑具备通水条件。通过国家卫生城市复审。

    科学推进新型城镇化。强化“一基本两牵动三保障”，全面落实城乡统一的户口登记制度。创新城镇化建设投融资机制，组建全省首家100亿元的浦银中建城市运营发展基金。林州市成为全国新型城镇化综合试点城市。

    促进城乡统筹发展。加快美丽乡村建设。改善农村人居环境走在全省前列，初步建成400个达标村、200个示范村。新改造农村危房3755户。解决39.6万农村居民饮水安全问题。硬化农村道路315公里。16个乡镇入围全省百强乡镇。

    （四）抓改革扩开放，着力激发经济发展活力。加快改革举措落地，着力提高开放招商实效，发展动能进一步释放。

    加快推进各项改革。深化行政体制改革，全面取消非行政许可审批，市政府部门全部建立权责清单，压缩行政职权1253项。完成市、县两级政府机构改革。建成全省首个网上“市民之家”。全面推行“三证合一”商事登记制度，新增市场主体3.7万户。稳步推进市区和林州市、汤阴县信用合作联社改制农商行。农村土地确权试点和市、县交通运输执法体制改革完成。党政机关公务用车改革顺利推进。财税体制、县级公立医院、价格等改革取得阶段成果。

    扩大开放招商。加强央企对接，与国旅集团、保利集团、中车集团等建立合作关系。与华强、恒大、建业等龙头企业合作进一步深化。积极融入京津冀协同发展战略，成功纳入“十三五”京津冀发展规划。组织参加“央企河南行”等重要招商活动。产业小镇、欧蓓莎环球港、广州童装（安阳）产业园等一批项目签约。全年引进省外资金520.7亿元，实际利用境外资金4.6亿美元。

    （五）惠民生增福祉，不断促进社会和谐。持续加大投入，十项民生工程56件实事圆满完成。

    实施精准扶贫。深入推进“115”扶贫增收计划，完成49个贫困村整村推进任务，2.9万人稳定脱贫。

    加强就业和社会保障工作。城镇新增就业8.2万人，农村劳动力转移就业136.8万人。提高低保、农村“五保”供养标准。全面建立临时救助制度。企业退休人员基本养老金“十一连增”。新开工保障性住房16202套，基本建成10862套。

    全面发展社会事业。扎实推进 “全面改薄”规划，贫困地区农村义务教育办学条件逐步改善。人均基本公共卫生服务经费补助标准达到40元，新农合和城镇居民医保人均财政补助标准提高到380元，市本级医保实现省内异地就医即时结算。积极推进文化惠民工程，文物保护工作得到加强。社会福利、慈善事业和养老服务业加快发展。全民健身运动蓬勃开展。依法保护妇女儿童合法权益。老龄、史志、档案、红十字和关心下一代工作取得新成效。

    着力防控和化解风险。建立债权银行联席会和贷款共进退机制，设立1亿元企业应急周转资金，有效化解企业资金链、担保链风险。建立健全工作机制，成立专案指挥部，有力有效处置非法集资。严格落实安全生产责任制，持续开展食品药品安全专项整治。加强社会治安综合治理，依法解决各类矛盾。

    一年来，我们扎实开展“三严三实”专题教育和“三查三保”活动，持续加强作风建设。强化行政监察和审计监督，开展懒政怠政、为官不为专项治理，不断健全权力运行制约和监督体系。自觉接受人大法律监督、政协民主监督和社会监督，认真办理人大代表议案、建议和政协提案。加强政务公开，推进服务型行政执法，法治政府建设取得新进展。扎实做好“双拥”工作，推动军民融合发展，军政军民团结更加巩固。加强老区建设。统计、气象、地震、邮政、发展研究、外事侨务、民族宗教等工作取得新成绩。

    各位代表！

    2015年各项目标的实现，标志着我市“十二五”规划胜利完成。五年来，面对外部环境复杂多变、结构调整阵痛加剧、下行压力持续增大、改革任务艰巨繁重的严峻形势，全市上下紧紧围绕建设区域性中心强市目标，全面落实党的十八大和十八届三中、四中、五中全会精神，深入贯彻习近平总书记系列重要讲话精神，主动适应经济发展新常态，真抓实干，克难攻坚，发展的科学性明显增强，后劲势能蓄积壮大，人民群众得到更多实惠，社会更加和谐稳定，为“十三五”发展奠定了坚实基础，全面建成小康社会站上了新的历史起点。

    ——综合实力不断提升。全市生产总值是2010年的1.5倍，年均增长8.7%；人均生产总值4.2万元，是2010年的1.5倍。规模以上工业增加值是2010年的1.7倍，年均增长11.2%。一般公共预算收入是2010年的1.6倍，年均增长10.1%。固定资产投资比2010年翻一番，年均增长15.5%。社会消费品零售总额是2010年的1.9倍，年均增长14.2%。

    ——发展方式加快转变。积极推进产业转型升级，高新技术产业增加值192.5亿元，年均增长15%，高于规模以上工业增加值增速3.8个百分点。产业集聚区固定资产投资年均增长19.1%，主营业务收入年均增长19.6%。文化旅游、电子商务等高成长性服务业快速发展，第三产业占比达到39.5%，提高11个百分点。土地规模经营比重达到32.4%。科技进步对产业发展的贡献率达到54%。单位生产总值能耗累计下降18%，超额完成“十二五”节能目标。

    ——城乡建设全面加强。常住人口城镇化率达到52%，较2010年提高13.4个百分点，高于全省5.7个百分点。林长高速、京港澳高速文峰立交桥等“六桥一互通”全部竣工。京广高铁、晋豫鲁铁路安阳段建成通车，“两纵一横”铁路枢纽基本形成。安阳机场前期取得实质进展。全市公路通车里程达到8297公里。成功创建全国绿化模范城市。

    ——改革开放逐步深化。重要领域和关键环节改革深入推进。市级行政审批事项大幅精简。商事登记制度、财税体制等改革加快推进。国企攻坚改革、集体林权制度改革完成。城乡居民大病保险、城乡居民基本养老保险、职业人群工伤保险制度实现全覆盖。开放载体平台加快建设，利用省外资金和实际利用外资分别是2010年的2.5倍和3.1倍，对外开放进入历史最好时期。

    ——人民生活持续改善。全市财政民生投入累计677.8亿元，占财政支出的74.3%。解决163.4万农村居民饮水安全问题。22.2万农村贫困人口稳定脱贫。城镇新增就业43.8万人，农村劳动力转移就业较“十一五”净增13.8万人。连续5年提高企业退休人员养老金和工伤保险待遇，连续两次调整失业保险金标准。城乡低保标准五年分别提高80%和116%，农村“五保”对象集中供养、分散供养标准分别提高90%和133%。累计建成保障性住房和棚户区改造住房85897套。科技、教育、文化、卫生等社会事业全面发展。

    各位代表！

    五年的历程很不平凡，取得的成绩来之不易。这是市委正确领导的结果，是市人大、市政协大力支持和监督的结果，是全市人民和衷共济、团结奋斗的结果。在此，我代表市人民政府，向奋斗在全市各条战线上的广大工人、农民、干部、知识分子，向解放军指战员、武警官兵、公安民警以及各界人士，致以崇高的敬意！向所有关心、支持我市现代化建设的港澳台同胞、海外侨胞、国际友人，表示衷心的感谢！

    总结成绩的同时，我们也清醒认识到，经济社会发展中还存在一些突出问题：一是经济下行压力增大，冶金建材、煤化工等能源原材料行业困难累积，企业投资预期下降；一些重大项目进展较慢，经济持续回升动力不足。二是产业结构不合理，质量效益不高；工业产品大多处于中低端，层次低、附加值低、成本高，市场竞争力不强。三是要素配置市场化程度低，制约明显加剧；高素质人才短缺，创新能力和科技成果转化能力弱；资本市场发育不充分，融资难问题突出，企业贷款担保链潜在风险增大。四是城市建设管理有待加强，公共服务水平低；教育、医疗、文化等民生领域还存在薄弱环节，历史欠账较多。五是部分区域环境承载能力超载，雾霾天气多发，制约了经济可持续发展。六是体制机制有待优化，一些审批事项环节多、耗时长，部门衔接不够、责任不清、办事效率不高。七是个别干部不适应新常态新要求，存在能力恐慌、精神疲弱、懒政怠政等现象。对此，我们一定高度重视，一一认真分析，找准症结，科学谋划，坚决行动，努力突破重围，努力让人民满意！

    二、“十三五”发展总体思路和主要目标

    “十三五”时期是全面建成小康社会的决胜阶段。综合分析，我市面临的外部环境依然复杂多变，但仍处于可以大有作为的重要战略机遇期，政策叠加优势日益凸显，综合竞争优势正在形成，改革开放红利加快释放，“四化”同步发展蕴含巨大潜力，干部群众思发展谋发展热情高涨，有利于我们抢抓机遇、战胜挑战，在新起点上实现转型发展、科学发展、永续发展。

    “十三五”时期经济社会发展的指导思想是：全面贯彻党的十八大和十八届三中、四中、五中全会精神，以马克思列宁主义、毛泽东思想、邓小平理论、“三个代表”重要思想、科学发展观为指导，深入贯彻习近平总书记系列重要讲话精神，按照“五位一体”总体布局和“四个全面”战略布局，坚持发展第一要务，牢固树立和贯彻落实创新、协调、绿色、开放、共享的发展理念，以提高发展质量和效益为中心，以转型升级为主线，深入实施开放带动、创新驱动和人才强市战略，加快形成引领经济发展新常态的体制机制和发展方式，打造安阳经济升级版、建设区域性中心强市，确保如期全面建成小康社会，开创建设富强安阳、创新安阳、文明安阳、平安安阳、美丽安阳新局面。

    主要预期目标是：经济保持较高速度增长，在提高发展平衡性、包容性、可持续性基础上，生产总值和城乡居民人均收入比2010年翻一番以上，力争主要人均指标达到全国平均水平。转型升级实现新突破，新型城镇化迈出新步伐，改革开放开创新局面，人民生活得到新改善，社会文明达到新水平，生态文明建设取得新成效。到2020年全面建成小康社会，力争综合实力重回全省第一方阵，区域性中心强市建设迈上新的台阶，现代化新安阳展现出更加美好前景！

    实现上述目标，贯彻五大理念，必须全面落实“11355”基本发展思路，即围绕全面建成小康社会、建设区域性中心强市一个目标，突出转型升级一条主线，实施开放带动、创新驱动、人才强市三大战略，夯实信息、交通、能源、水利、生态五大基础，强化科学发展载体、现代产业体系、现代城镇体系、现代创新体系、现代市场体系五大支撑，确保“十三五”各项目标任务圆满完成。

    根据市委建议，市政府编制了《安阳市国民经济和社会发展第十三个五年规划纲要（草案）》，已印发会议，提请审议。

    各位代表！

    功崇惟志，业广惟勤。实现安阳振兴，创造全市人民更加美好的生活，任重而道远。我们坚信，经过五年的不懈奋斗，全面建成小康社会的宏伟蓝图一定能够如期实现！

    三、2016年重点工作

    今年是“十三五”规划的开局之年。做好今年政府工作，必须全面落实市委十届十七次全会部署，适应经济发展新常态，加强供给侧结构性改革，去产能、去库存、去杠杆、降成本、补短板，坚持“一个引领”，突出“六大举措”，实施“六大工程”，就是以习近平总书记关于“经济新常态怎么看、怎么干”的重要论述为引领，突出项目拉动、开放带动、创新驱动、改革牵动、“四化”联动、人才推动，大力实施“461”项目推进工程、文化旅游提升工程、棚户区改造工程、“互联网+”行动推进工程、生态环境整治修复工程、脱贫攻坚工程，牢牢把握工作主动权。

    今年经济社会发展的主要预期目标是：生产总值增长7.5%左右，在实际工作中争取更好结果；一般公共预算收入增长6.5%，全社会固定资产投资增长15%左右，社会消费品零售总额增长12%，进出口总值增长7.5%，实际到位省外资金增长7%，城镇新增就业6万人以上，常住人口和户籍人口城镇化率均提高1.6个百分点，城乡居民收入增长与经济增长基本同步，居民消费价格涨幅3%左右，节能减排、环保等约束性指标完成省下达任务，发展质量和效益明显提高，结构性改革取得实质性进展。重点抓好九个方面工作：

    （一）抓好结构性改革，加快产业提质转型。坚持供需两端发力，突出供给侧结构调整，为稳定增长、提质发展提供强劲动力。

    提升产业竞争力。以加快新旧产业动力转换为中心任务，以需求特别是消费升级趋势为导向，强化工业化信息化融合，推进“工业转型升级400”，启动实施千亿投资计划，优化服务业结构，壮大服务业规模，推动产业水平整体提升。一是发挥特色优势，大力发展先进装备制造业。支持重大技术攻关、骨干企业智能化改造和重大产业基地建设，推动数控机床、锻压设备、矿山装备、精密制造、智能机器人、航空植保无人机等优势领域率先突破。二是降本增效、整合重组，大力推动冶金、煤化工行业转型。支持安钢等龙头企业实施战略重组，加快产品结构调整，延伸补强下游精深加工产业链。规划建设精细化工产业园。支持安化等煤化工企业实施循环化改造。三是推动集群发展，大力增创食品医药行业优势。支持永达、众品、益海嘉里、科伦药业等骨干企业提升研发能力，培育知名品牌，壮大产业集群，增强行业竞争力。四是加快整合提升，大力推进纺织服装产业集聚发展。以中原高新技术开发区、彰北针织服装产业园和柏庄纺织产业园为重点，引进国内外行业龙头企业。加快染整产业园规划建设，完善纺织产业链条。五是瞄准主攻方向，大力培育壮大电子信息产业。加快建设产业小镇，重点承接电子基础材料、电子传感器、电子元器件、高端电子玻璃、新型显示器件、家电灯饰等产业蜂窝式转移。六是抓好推广应用，大力发展节能环保和新能源汽车产业。开展节能环保产品推广示范，鼓励支持艾尔旺、利浦筒仓、方快锅炉等企业加强先进节能环保技术、工艺和装备研发。积极配建充电设施，完善低速电动车规范运营机制。七是强化示范引导，大力发展都市生态农业。围绕“两圈两岭三区三带”，推进现代农业园区示范创建，重点发展休闲观光、农耕体验、生态创意等新业态。八是突出现代物流、现代金融，大力发展生产性服务业。完善物流体系，推进物流园区智能化、信息化和标准化建设。抓好安阳保税物流中心、天津自贸区安阳分区、天津港安阳无水港、安阳铁路口岸等建设。推动金融集聚区建设。加快培育信息、人才、科技、中介等服务机构。九是实施“互联网+”行动推进工程，大力发展新业态、新模式。制订“互联网+”11个行动计划和39个重大专项。加快完善 “一中心、三平台”。加强中部易商谷等电子商务产业园建设，发展跨境电商。提升健康养老、教育培训、医疗保健、家政服务、文化体育等服务消费能力。十是去库存、促消费，大力促进房地产业健康发展。探索建立购租并举的住房制度，提高棚改货币化安置率。鼓励农民进城购房，将有稳定劳动关系的常住人口纳入公积金覆盖范围。

    强化企业服务。一是提高帮扶实效。建立企业服务台账，每月组织开展重点企业专项巡回服务活动。抓好产销、银企、用工、产学研“四项对接”。落实好国家和省减轻企业负担各项措施。二是创新政策支持方式。用好国家、省和我市各类基金，采取购买服务、股权投资等方式，支持领军企业发展和优质项目建设。进一步放宽服务业准入门槛。三是培育科技小巨人企业。重点支持合力冷轧、中农颖泰、全丰农飞客等33个具有核心技术的中小企业发展，培育新的经济增长点。四是积极稳妥处置各类困难企业。对长期亏损、资不抵债、停产半停产企业，鼓励兼并重组或依法破产，妥善安置职工、处置债务，有序实现市场出清。对不符合能耗、环保、质量、安全等标准的企业，推动提标改造、优化升级。对产品有市场、有效益但暂时遇到困难的企业，继续予以支持。

    完善科学发展载体。加强优势产业集群培育，完善考核评价体系，推进产业集聚区和服务业“两区”管理创新，加快构建与乡镇行政区域管理套合的管理体制。力争全年产业集聚区完成投资750亿元，实现工业总产值1800亿元。

    （二）抓好“461”项目推进工程，加快扩大有效投资。把项目建设作为全年工作主抓手，按照竣工、在建、前期进行分类管理，确保按时竣工、按节点推进、按期开工建设。力争220个重大项目超额完成投资计划。

    抓好70个重大工业升级项目。确保安钢120万吨冷轧薄板、安阳精密制造产业基地模范工厂、中海清华智能机器人产业园（一期）、翔宇医疗康复设备产业园等一批项目竣工投产，加快国家863红旗渠科技产业园、德力新能源专用汽车、华德重工汽车零部件等一批项目建设，力争全年完成投资163亿元。

    抓好85个重大服务业项目。确保长德商贸物流城、泰祥汽博产业园等一批重大项目竣工运营，加快国旅安阳城、内黄果蔬城、万庄物流园、欧蓓莎环球港等一批项目建设，力争全年完成投资210亿元。

    抓好65个重大交通能源项目。重点推进西北绕城高速、国道341、新东北外环、林州新创光伏电站、华润新能源、华能汤阴风电场等重大交通设施和新能源项目建设，力争全年完成投资113亿元。加快林焦高速林州段、安林市域铁路、高铁站客运枢纽、安阳机场等项目前期工作，力争开工建设。

    （三）抓好新型城镇化建设，加快释放发展潜力。强化新型城镇化带动，促进城乡统筹、区域协调，拓展发展空间。

    有序推进农业转移人口市民化。加快户籍制度改革和居住证制度“双落地”，维护好进城落户农民土地承包权、宅基地使用权、集体收益分配权。

    推动中心城区和城市组团协调发展。坚持“1+4+1”城市发展格局，推进中心带动、错位发展、联动发展。加快市域铁路、快速通道、旅游专线、产业园区规划建设，促进中心城区与城市组团交通一体、产业链接、功能互补、生态共建、良性互动。

    放手放权发展中心城区经济。坚持工业主导、三业兴城，优化产业布局，探索利益分享、园区共建、飞地经济等发展模式，促进重大产业项目向城区集聚。支持各区发挥比较优势，明确功能定位，集中要素资源，以商贸物流、文化旅游为重点，打造生产性服务业集聚区、生活性服务业精品区。加快宝莲寺高端商务区、龙泉生态文化旅游休闲度假区、殷都区金融产业集聚区、洹河两岸文化旅游开发项目建设。支持城乡一体化示范区、高新区建设重大产业基地，加强与天津滨海新区合作，提升对外开放水平，引领城区经济发展。

    强化城镇规划建设管理。加强空间管制，严格落实城市总体规划，做好土地利用总体规划调整和永久基本农田划定工作。加快编制县级城市城乡总体规划、村庄规划和城市近期建设规划。全面推广城市管理“十大规矩”，巩固提升国家卫生城市复审成果。加快兴业路等18条小街小巷建设。完成文昌大道、永明路南北段征地拆迁。完成平原路南段及南林高速下穿工程和60项保障性安居工程配套基础设施建设。开工建设光明路南林高速下穿工程。启动建设新垃圾处理厂。完善城区供热、供水、供电、防灾等基础设施。积极推进城市综合管廊和海绵城市试点建设。

    加强历史文化名城保护建设。坚持政府主导、市级为主、市区联动、市场运作、社会参与的原则，科学制定保护建设规划。建立健全工作机制，多元融资，分类推进，启动老城保护建设、护城河治理、文化遗迹保护修缮等工程。制订实施重点历史文化街区、历史文化村镇保护建设行动计划。推动渔洋村等7个国家级、西蒋村等5个省级传统村落保护建设和提升。

    大力实施棚户区改造工程。强化推进机制，细化目标任务，严格奖惩措施，确保26个棚户区改造项目按时开工、按期完成。

    统筹城乡协调发展。加强水冶镇、宜沟镇、临淇镇等重点镇和特色镇建设，完善发展规划，发展特色产业，建立考评体系，确保完成阶段任务。加快城镇基础设施和公共服务设施向农村延伸，推进农村人居环境综合治理。完成美丽乡村建设任务。

    （四）抓好稳粮提质增效，加快发展现代农业。坚持集约高效绿色可持续发展，推进农业供给侧结构性改革，构建现代农业产业体系、生产体系、经营体系，建设现代农业示范市。

    推进高标准粮田建设。坚持藏粮于地、藏粮于技，强化技术集成推广，整合各类农业资金，新建高标准粮田30万亩，确保粮食总产稳定在220万吨以上。

    壮大农业产业集群。加强林州市、内黄县、安阳县农产品专业加工园区和汤阴县农产品综合加工园区建设，扩大五大基地规模，重点提升15个10亿元集群发展水平，形成50亿元集群1个、20亿元集群3个，集群总收入达到280亿元。

    深化现代农业园区示范创建。推进内黄县都市生态、汤阴县汤河、安阳县环小南海库区、林州市红旗渠等现代农业综合园区和专业园区建设，强化示范引导，促进现代农业园区和都市农业融合发展。新建扩建园区30个，园区总面积达到85万亩。建设市级示范园区10个。

    创新完善农业经营体系。推进土地适度规模经营，加快培育新型农业经营主体，实施新型职业农民、现代青年农场主培育计划，农民合作社达到3500个，家庭农场达到1000家。抓好特色区域站创建、农业科技创新和应用推广，提升主要农作物全程机械化水平。推进蔬菜瓜果、花卉苗木、农资、农机、冷链物流等骨干工程建设。打造农业电商品牌。

    加强农业生态保护。推广节约农业技术，抓好农业面源污染整治和耕地地力提升。完善农产品质量安全监管体系，推进农业标准化生产和可追溯试点建设。抓好大中型沼气工程项目，发展循环生态农业。

    （五）抓好文化旅游提升工程，加快提升产业质量。发挥文化旅游资源优势，强化政策支撑和责任落实，着力建设华夏历史文化传承创新市和国际旅游城。

    突出品牌打造。全面整合旅游资源，集中力量打造山水风光游精品线路，以山水安阳带动文化安阳、历史安阳。加快培育航空运动旅游、乡村旅游等消费热点。建立智慧旅游服务网络。加强旅游行业管理和服务培训，塑造旅游服务新形象。

    突出旅游推介。围绕京津冀、韩国等重点区域，充分利用网络等新兴媒体，加强高水平策划，全方位抓好旅游宣传。强化与旅行商对接合作，深度拓展旅游客源市场。办好韩国万人游安阳、汉字听写大会、航空运动文化旅游节等活动。力争全年接待国内外游客3800万人次。

    突出基础建设。完善旅游标识系统、游客服务中心、集散中心、旅游厕所等配套服务设施，加强主要景区连接交通干线的旅游公路建设。做好中国文字博物馆续建等项目前期工作。加快推进保利影视文化广场、职工文化体育中心、妇女儿童活动中心、红旗渠文化旅游产业园等项目建设。加强文物和非物质文化遗产保护，推进红旗渠申报世界文化遗产。

    突出机制建设。成立旅游发展委员会，建立完善文化旅游管理机制。编制旅游业发展规划。完善文化设施免费开放保障机制。加强文化广电新闻出版管理。推进有线电视数字化改造。建立文化旅游发展基金，支持文化产品研发和重点项目建设。

    （六）抓好创新驱动，加快提升发展动力。把创新放在发展全局的核心位置，用好创新发展基金，着力打造创新驱动新引擎。2016年，高新技术产业增加值占规模以上工业增加值比重达到30%左右，高成长性制造业投资占工业投资的比重超过50%。

    完善创新创业载体。优化“双创”政策环境。加快市民之家众创空间、赛普工业研究院、科创大厦、国家农业科技示范园区、安阳综合检测基地暨国家铁合金质检中心等创新平台建设。

    提高企业创新能力。推动大中型企业研发中心全覆盖。加强与清华大学、西安交大、合肥工大等高校合作。新建一批重点实验室、工程技术中心、创新联盟。培育10家国家级高新企业。加强信用体系建设，强化知识产权保护，着力培育知名商标。

    加强科技对外合作。开展“百家院校科技成果走安阳”活动。实施10个以上合作项目，引进10个以上国内外科技成果，启动实施3个重点领域科技专项。

    实施人才强市战略。建立人才开发基金，对引进的高层次人才和高水平研发团队，加大扶持力度。完善科技成果参与收益分配和奖励政策，探索“引项目+引技术+引人才”新模式，促进人才集聚。支持高等院校内涵式发展，推动安阳工学院向应用型本科院校转型。提高职业学校办学水平，重点支持3所河南省职业教育品牌示范院校和6所特色院校建设。

    （七）抓好开放带动，加快推进各项改革。坚定不移促改革、扩开放，着力提高改革开放实效，释放发展动力活力。

    打造区域开放新高地。主动融入国家战略。围绕 “一带一路” 、京津冀协同发展战略和我省三大国家战略的重大投资走向，主动加强与国家有关部委对接，促进重大产业和项目转移落地。围绕我市列入京津冀周边区域协同联动发展范围，重点规划建设“两区五基地”。加快推进前期谋划对接的50个重大项目。推动外贸进出口稳定增长，鼓励有条件的企业“走出去”。创新招商方式。围绕重点区域和重点对象，加强产业链招商、集群招商、精准招商，大力承接长三角、珠三角、京津冀等地区产业转移。组织参加中国（河南）国际投资贸易洽谈会、豫商大会、中国郑州产业转移专项对接等活动。充分发挥行业协会、商会作用，着力引进企业总部入驻。狠抓招商项目落地。强化签约项目专项督查，建立项目前期工作责任制，确保合同履约率、资金到位率和项目开工率居于全省前列。

    持续深化改革。继续简政放权。精简行政审批事项，加快行政审批标准化建设。取消不合法不合理的收费和基金项目，完善收费公示制度，确保各项改革接得住、放得下、管得好。编制公开县级政府权力清单和责任清单。扩大“三证合一”改革成果。深化国资国企改革。研究制定新一轮国资国企改革实施意见，努力解决豫北纱厂、黄河轮胎、平原制药等企业改制遗留问题。整合建立统一的公共资源交易平台。全面清理限制非公有制经济发展的不合理规定。探索建立企业投资项目负面清单。深化投融资体制改革。推进营改增扩围、消费税改革和资源税费改革。继续实施政府购买公共服务。加快市级投融资公司改革。加强企业上市辅导，争取30家企业在新三板等各类股权交易场所挂牌融资。深化农业农村发展改革。基本完成农村土地确权登记颁证工作。深化供销社综合改革。抓好安阳县全国农村承包土地经营权抵押贷款试点、林州市农村产权交易中心试点。创新农业保险、农村金融服务产品。深化管理体制改革。科学调整行政区划。理顺城乡一体化示范区管理体制机制。加强经济发达镇行政管理体制改革试点工作。同时，加快推进综合行政执法体制、事业单位、公立医院、价格等改革。

    （八）抓好生态环境整治修复工程，加快改善人居环境。坚持发展、保护并重，多措并举，全面加强生态保护。

    坚持铁腕治污。实施蓝天工程，继续深入开展大气污染防治，全面建立控煤、控尘、控烟、控油、控车责任制，确保大气质量明显改善、优良天数明显增加。实施碧水工程，重点抓好入河排污口截流工作，确保省控出境断面达标率符合省定目标。完善垃圾、污水收集处理配套基础设施。

    筑牢生态安全屏障。抓好太行山绿化、平原林网、通道绿化、城郊绿化以及森林公园、湿地公园建设，构建“1+3+N”林业生态体系。

    加强水生态体系建设。落实最严格水资源管理制度，抓好城乡防汛、河道治理。加快推进引岳入安、引红入洹、引彰入汤等水资源调配和水系联通工程。实施万金渠城区段清淤工程和灌区续建配套工程。做好“宝莲湖”前期。编制城市水系综合利用景观规划、洪河治理保护规划，着力打造城市水系景观。

    完善生态保护机制。强化生态保护红线管理制度和污染防治区域联动机制，建立健全覆盖所有固定污染源的企业排放许可、评价考核与责任追究制度。探索主要污染物排污权有偿使用和交易试点收费改革。推广清洁能源，突出抓好矿产资源综合利用和循环利用，推动绿色、低碳、循环发展。

    （九）抓好民生改善，加快实现共享发展。以实施民生工程为重点，加快健全基本公共服务，维护社会和谐稳定。

    大力实施脱贫攻坚工程。强化精准扶贫、精准脱贫，落实脱贫攻坚领导责任制，坚持“转、扶、搬、保、救”五措并举，强力推进“3456”脱贫攻坚行动计划，确保2.8万贫困人口脱贫。

    稳定扩大就业。坚持就业优先，推动大众创业，以创业带动就业。实施大学生就业促进计划和大学生创业引领计划。强化对困难人员的就业援助，支持农村劳动力转移就业和返乡农民工创业。确保实现城镇新增就业目标，实现农村劳动力转移就业136.5万人。

    增加城乡居民收入。健全企业工资集体协商、正常增长和支付保障机制。适当提高机关事业单位人员基本工资。落实县以下机关公务员职务与职级并行制度和乡镇工作补贴。多渠道增加农民收入。依法治理拖欠农民工工资问题。

    强化社会保障。实施全民参保登记计划。继续提高企业退休人员基本养老金。完善城乡居民基本医疗保险筹资机制。新农合和城镇居民医保年人均补助标准再提高30元。提高城乡低保、医疗救助、临时救助保障水平，城乡低保财政补差水平年人均分别提高180元和144元，农村“五保”对象年集中供养、分散供养标准分别提高到人均4000元和3000元。建立困难残疾人生活补贴、重度残疾人护理补贴和政府购买养老服务制度。健全农村“三留守”人员关爱服务体系，建立经济困难高龄失能老人补贴制度。筹建市残疾人综合服务中心。完成县级社会福利中心建设。扎实做好老龄、慈善、红十字等工作。

    发展社会事业。促进义务教育均衡发展，强力实施“全面改薄”项目，推进城镇教育资源扩充。抓好市六十八中、文峰区明德小学等12所中小学和44个幼儿园新建或改扩建工作。免除建档立卡家庭经济困难普通高中学生学杂费。支持和规范民办教育发展。筹建市级公共卫生服务中心，确保市人民医院搬迁、妇幼保健院儿科综合楼、第三人民医院门诊综合楼、疾控中心食安设备项目建成投用。加快乡镇卫生院、社区卫生服务机构标准化建设。发展智慧医疗，实施健康安阳促进行动，全面推行卫生计生融合“七证合一”服务模式，做好全面“两孩”政策的实施。健全公共文化服务体系，开展“321”公共文化共建活动，持续实施重点文化惠民工程，加快基层综合性文化服务中心建设。大力发展体育事业，广泛开展全民健身运动，继续实施老年体育长青工程。

    创新社会治理。健全社会治安立体防控体系，强化流动人口和特殊人群服务管理。做好信访维稳工作，注重依法化解社会矛盾。深入开展处置非法集资攻坚年活动，力争年内取得重大突破。用好应急周转资金，完善债权银行联席会和贷款共进退机制，有效化解企业担保链风险。严格落实安全生产责任制，加强安全监管执法，坚决遏制重特大事故发生。完善食品药品安全监管体制机制，确保不发生重大食品安全事故。

    大力弘扬焦裕禄精神和红旗渠精神，培育和践行社会主义核心价值观。深入推进公民道德建设，提升全民文明素养，打造文明安阳。支持国防和军队改革，支持驻安部队建设发展，积极推进民兵预备役建设，深入开展“双拥”共建，推动军民融合深度发展。加强老区建设。支持工会、共青团、妇联等人民团体发挥桥梁纽带作用。认真做好关心下一代工作。开展第三次全国农业普查。继续做好史志、档案、气象、地震、邮政、发展研究、外事侨务、民族宗教等工作。

    四、加强政府自身建设

    加快安阳发展，人民寄予厚望，政府责任重大。必须深入贯彻法治政府建设实施纲要，全面打造法治、服务、高效、廉洁政府，持续提升政府执行力和公信力。

    （一）重法治、严规范，依法行政。坚持依法治市，建立健全行政立法机制和依法决策机制，完善公众参与、专家论证、风险评估、合法性审查等重大行政决策法定程序。实施行政执法责任制，加强行政执法监督，推进服务型行政执法。启动“七五”普法。依法接受人大及其常委会法律和工作监督，主动接受政协民主监督。凡是市委的决策、人大的决议，坚决贯彻执行到位；凡是人大代表议案、建议和政协提案，坚决依法妥善办理；凡是政府的重要安排、部门的主要工作，及时向人大报告、向政协通报。广泛听取民主党派、工商联、无党派人士、人民团体意见，自觉接受社会监督和舆论监督，确保权力在阳光下运行！

    （二）勇担当、真作为，改进作风。认真践行“三严三实”，深入开展作风建设年活动，扎实推进懒政怠政、为官不为专项治理，转政风、正行风、刹“四风”。坚持问题导向、目标导向、改革导向、责任导向，少说多做、埋头苦干，多一线调研、接地气，多现场办公、解难题。坚持以上率下，层层看齐，立标杆，定规矩，作示范，用行动改变安阳，用行动发展安阳，用行动振兴安阳！

    （三）建机制、抓落实，高效运作。按照“三准三专三聚三提”要求，对每一项重点任务、重点项目、重点工程、重点举措进行细化分解，建台账、明责任、严督察，挂图作战，一抓到底，干出成效。完善项目在线审批监管平台，健全岗位履职责任制，建立全覆盖大督察机制，强化纠错问责，坚决打通政策落实 “中梗阻”和“最后一公里”！

    （四）筑防线、守规矩，廉洁从政。坚持把纪律和规矩挺在前面，履行主体责任，强化“一岗双责”，全面落实党风廉政建设责任制。加强廉政教育，严格执行廉洁自律准则和各项制度规定，强化行政监察和审计监督，坚决查处发生在群众身边的腐败问题。坚持无禁区、全覆盖、零容忍，有腐必惩、有贪必肃，以反腐倡廉实效保障发展、取信于民！

    各位代表！

    真抓才能攻坚克难，实干才能梦想成真。舟至中流，更需击楫勇进。让我们紧密团结在以习近平同志为总书记的党中央周围，在市委的坚强领导下，以更加扎实的作风，更加饱满的热情，更加昂扬的斗志，开拓创新，砥砺前行，夺取全面建成小康社会新胜利，谱写建设区域性中心强市新篇章！
